# The association of intensive care with utilization and costs of outpatient healthcare services and quality of life

**DOI:** 10.1371/journal.pone.0222671

**Published:** 2019-09-20

**Authors:** Robert P. Kosilek, Sebastian E. Baumeister, Till Ittermann, Matthias Gründling, Frank M. Brunkhorst, Stephan B. Felix, Peter Abel, Sigrun Friesecke, Christian Apfelbacher, Magdalena Brandl, Konrad Schmidt, Wolfgang Hoffmann, Carsten O. Schmidt, Jean-François Chenot, Henry Völzke, Jochen S. Gensichen

**Affiliations:** 1 Institute of General Practice and Family Medicine, LMU München, Munich, Germany; 2 Chair of Epidemiology, LMU München, UNIKA-T Augsburg, Augsburg, Germany; 3 Independent Research Group Clinical Epidemiology, Helmholtz Zentrum München, German Research Center for Environmental Health, Munich, Germany; 4 Institute for Community Medicine, University Medicine Greifswald, Greifswald, Germany; 5 Department of Anesthesiology, University Medicine Greifswald, Greifswald, Germany; 6 Integrated Research and Treatment Center Center for Sepsis Control and Care (CSCC), Jena University Hospital, Jena, Germany; 7 Department of Internal Medicine B, Medical Intensive Care Unit, University Medicine Greifswald, Greifswald, Germany; 8 DZHK (German Center for Cardiovascular Research), Partner Site Greifswald, Greifswald, Germany; 9 Medical Sociology, Institute of Epidemiology and Preventive Medicine, University of Regensburg, Regensburg, Germany; 10 Family Medicine and Primary Care, Lee Kong Chian School of Medicine, Nanyang Technological University Singapore, Singapore; 11 Institute of General Practice and Family Medicine, Charité University Medicine Berlin, Berlin, Germany; 12 Institute of General Practice and Family Medicine, Jena University Hospital, Jena, Germany; 13 German Center for Diabetes Research, Site Greifswald, Greifswald, Germany; East Carolina University Brody School of Medicine, UNITED STATES

## Abstract

**Background:**

Little is known about outpatient health services use following critical illness and intensive care. We examined the association of intensive care with outpatient consultations and quality of life in a population-based sample.

**Methods:**

Cross-sectional analysis of data from 6,686 participants of the Study of Health in Pomerania (SHIP), which consists of two independent population-based cohorts. Statistical modeling was done using Poisson regression, negative binomial and generalized linear models for consultations, and a fractional response model for quality of life (EQ-5D-3L index value), with results expressed as prevalence ratios (PR) or percent change (PC). Entropy balancing was used to adjust for observed confounding.

**Results:**

ICU treatment in the previous year was reported by 139 of 6,686 (2,1%) participants, and was associated with a higher probability (PR 1.05 [CI:1.03;1.07]), number (PC +58.0% [CI:22.8;103.2]) and costs (PC +64.1% [CI:32.0;103.9]) of annual outpatient consultations, as well as with a higher number of medications (PC +37.8% [CI:17.7;61.5]). Participants with ICU treatment were more likely to visit a specialist (PR 1.13 [CI:1.09; 1.16]), specifically internal medicine (PR 1.67 [CI:1.45;1.92]), surgery (PR 2.42 [CI:1.92;3.05]), psychiatry (PR 2.25 [CI:1.30;3.90]), and orthopedics (PR 1.54 [CI:1.11;2.14]). There was no significant effect regarding general practitioner consultations. ICU treatment was also associated with lower health-related quality of life (EQ-5D index value: PC -13.7% [CI:-27.0;-0.3]). Furthermore, quality of life was inversely associated with outpatient consultations in the previous month, more so for participants with ICU treatment.

**Conclusions:**

Our findings suggest that ICU treatment is associated with an increased utilization of outpatient specialist services, higher medication intake, and impaired quality of life.

## Introduction

Over the past decades, intensive care unit (ICU) treatment has become more effective, and the related inpatient and post-discharge mortality has declined in several Western countries. [1] However, this was associated with a growing number of patients suffering from long-term physical and neuropsychiatric impairments, which were recently summarized under the term postintensive care syndrome (PICS). [1, 2] While the exact prevalence of PICS is unknown, it is estimated that associated impairments occur in at least 1 of 4 survivors of critical illness and intensive care. [3–5] Short- and long-term impairments in quality of life and a significant socioeconomic burden in survivors of critical illness have previously been demonstrated. [6–8] The evidence regarding post-ICU follow-up strategies is conflicting—a recent systematic review and meta-analysis has found that the overall quality of evidence was low, and that follow-up interventions did not demonstrate any relevant effect on quality of life. [9] Several studies have shown that ICU treatment is associated with increased healthcare resource utilization and costs. [10–18] However, there are only few studies on the associated utilization of outpatient health services, specifically specialist consultations. [18] The German healthcare system consists of statutory public health insurance with mostly free choice of treatment providers, which offers a good opportunity to examine the use of healthcare services by ICU survivors. [19] Therefore, we used data from a German population-based study, the Study of Health in Pomerania (SHIP), to examine the association of ICU treatment with outpatient health services utilization, costs, and health-related quality of life.

## Subjects and methods

### Study design and population

SHIP consists of two independent cohorts. It is a population-based study of adult residents of West Pomerania in northeastern Germany between 20 and 79 years of age. The study design, protocol and sampling methods have been described in previous publications. [20, 21] It was approved by the ethics committee of the University of Greifswald and adheres to the Declaration of Helsinki. All study subjects gave written informed consent prior to participation. This study is reported according to the Strengthening the Reporting of Observational Studies in Epidemiology (STROBE) recommendations. [22] For the first cohort, 4308 out of 6265 eligible individuals participated at the baseline examination (SHIP-0) between 1997 and 2001. The first follow-up at five years (SHIP-1) was conducted between 2002 and 2006 with 3300 participants. The second follow-up at ten years (SHIP-2) was conducted between 2008 and 2012 with 2333 participants. For the second independent cohort (SHIP Trend), 4420 out of 8826 eligible individuals participated in the baseline examination (Trend-0) between 2008 and 2012. Data from the examinations SHIP-2 and Trend-0, both conducted between 2008 and 2012 with a comparable study design and identical measurements, were thus used for a pooled cross-sectional analysis. Out of a total sample of 6.753 individuals, 67 were excluded due to missing interview data on healthcare services utilization, resulting in a final analytical sample of 6.686 subjects. Data from SHIP-0 and SHIP-1 were not used for analyses because the exposure of interest (ICU treatment) was not assessed until SHIP-2.

### Data

Information on socioeconomic characteristics, lifestyle habits, medical history, medication use, somatometric measures, blood pressure, and health services utilization was gathered by trained study staff during standardized examinations and interviews. [21]

### Health services utilization and costs

Inpatient health services utilization was assessed by asking for the number and duration of hospital treatments in the previous 12 months. Participants were additionally asked if they had received ICU treatment during this time, which served as the key exposure variable for our analyses. Outpatient health services utilization was assessed by asking which types of physicians from a list of 12 common specialties were consulted in the previous year. Study participants could additionally name specialist consultations that were not covered by the list. These responses were reassigned to any of the listed categories if possible (e. g. cardiologist/internal medicine), and otherwise included in calculations as a specialist visit. The analyses were restricted to general outpatient health services and excluded visits to dentists. Only in SHIP-2, subjects were additionally asked to report the number of consultations in the previous year. Analyses regarding the number and costs of consultations were therefore restricted to this cohort. An exception to this is the total number of consultations in the previous four weeks, which was asked for in SHIP-2 and Trend-0 as a separate question. The number of current medications excluding contraceptives, classified by ATC code, was used as an additional indicator of healthcare resource utilization. Direct medical costs from a societal perspective were calculated based on a bottom-up micro-costing approach, according to recommendations of the German Working Group on Methods in Health Economic Evaluation and standardized unit costs for Germany from Bock et al. [23, 24] Specific standard cost rates were applied to the type and number of consultations (e. g. 20.06 € per general practitioner visit) and inflated using the consumer price indices for health care in Germany from 2008 to 2012.

### Health-related quality of life

The EuroQol EQ-5D-3L quality of life instrument was used to assess health-related quality of life. [25] It is designed for self-completion by the respondent and captures the health status according to the respondent’s situation at the time of completion. The instrument has been validated for several countries, resulting in country-specific general population value sets. [26] Individual responses on the five EQ-5D subdomains (mobility, self-care, usual activities, pain/discomfort, anxiety/depression) were used to calculate the EQ-5D index value with value sets for Germany using Stata’s *eq5d* package. [27] The EQ-5D index value is a preference-based valuation of health-related quality of life, and ranges from 0 (death) to 1 (best health).

### Control variables

We controlled for several baseline characteristics that were assumed to affect health services utilization and quality of life. Control variables were selected according to Andersen’s Behavioral Model of Health Services Use that emphasizes contextual as well as individual determinants of access to medical care. [28, 29] We assumed that direct causes of the exposure or outcome, and exclusion of possible instrumental variables that affect the outcome only through the exposure, is a valid criterion to identify a sufficient set of controls. [30] We included age, gender, body-mass-index, waist-to-height ratio, relationship status, health insurance type, education (completed school years) and equivalent household income (calculated from annual income and household size according to the Luxembourg Income Study recommendation [31]), smoking status (never, current, former), alcohol consumption in grams of ethanol per day (beverage-specific quantity-frequency measure [32]), and physical inactivity defined as less than 1 hour of physical activity per week during summer and winter months. Comorbidity was assessed using the number of selected present chronic conditions that commonly occur in critically ill patients: cardiovascular (hypertension, myocardial infarction, stroke), pulmonary, kidney and liver disease, diabetes, cancer. [33]

### Statistical analyses

Stata 15.1 was used for statistical analyses (Stata Corp., College Station, TX, USA).

#### Adjustment for drop-out and confounding

We used inverse probability weighting to address drop-out from SHIP-0 to SHIP-2; subjects from Trend-0 were assigned a probability weight of 1. A logistic model that included socio-economic, behavioral and health-related predictors was used to derive stabilized inverse probability weights. [34] Entropy balancing (as implemented in the Stata package *ebalance* [35]) was used to adjust for confounding. This method reweights comparisons groups (i.e. by ICU treatment status) to make them comparable on measured control variables (Table A in S1 Appendix). [36] We assessed the validity of analytical weights according to published balance diagnostics in propensity score analysis, with standardized differences greater than 10% indicating risk of bias. [37] We further assessed how substantial unmeasured confounding would need to be to explain away the observed associations by calculating the E-value for regression estimates (Tables B and C in S1 Appendix). [38] Regression models included the weights obtained from entropy balancing, and were additionally adjusted for age, gender, the sum of comorbidities and a study indicator variable (SHIP-2 vs. Trend). There were less than 1% missing values and these were imputed. For EQ-5D analyses, we excluded participants that did not provide any answers on the EQ-5D questionnaire by listwise deletion (n = 18, 0.27%).

#### Regression analyses

We used Poisson regression models with robust standard errors to estimate prevalence ratios (PR) for any outpatient consultations, medication intake and impairment in EQ-5D subdomains. [39] A negative binomial regression model was used to estimate the number of consultations and current medications. A generalized linear model with gamma-distribution and a log-link function was used to estimate consultation costs. [40] Effect estimates from these models were expressed in terms of percent change (PC) compared to the reference group of participants without ICU treatment. The EQ-5D index value ranges from zero to one with a left-skewed distribution. We used a fractional response model to accommodate the features of this outcome variable; effects were expressed as PC in terms of average marginal effects. [41] We provided 95% confidence intervals (CI) for all effect estimates.

## Results

Baseline characteristics of the study population are reported in Table 1. As expected, the distribution of baseline characteristics differed across groups. ICU treatment in the previous year was reported by 139 of 6686 subjects (2.1%). Compared to subjects with no ICU treatment, post-ICU subjects were older (median age 64 vs. 54 years), predominantly male (67.6% vs. 47.6%), and had a higher prevalence of comorbidities (any comorbidity: 92.8% vs. 72.3%), among other aspects. After applying balancing weights, we found no standardized differences greater than 10% (Table A in S1 Appendix), which underscored that groups were comparable after conditioning on the control variables.

**Table 1:** Baseline characteristics of the study population by ICU treatment status.

Variable	ICU treatment in previous 12 months					
	No (97.9%)			Yes (2.1%)		
	(N = 6,547)			(N = 139)		
Data Source* (SHIP Trend-0)	**65.3%**	/	*4274*	**63.3%**	/	*88*
Gender (Male)	**47.6%**	/	*3116*	**67.6%**	/	*94*
Age, years	**54.0**	/	*23*.*0*	**64.0**	/	*24*.*0*
Education, school years	**11.0**	/	*2*.*0*	**11.0**	/	*3*.*0*
Equivalent household income, €	**1183.6**	/	*866*.*0*	**1096.0**	/	*671*.*8*
In relationship	**77.8%**	/	*5093*	**80.6%**	/	*112*
Physical inactivity	**31.3%**	/	*2046*	**28.8%**	/	*40*
Body mass index, kg/m^2^	**27.5**	/	*6*.*5*	**29.2**	/	*6*.*7*
*Body mass index* ≥ *30 kg/m^2^*	**31.6%**	/	*2068*	**42.5%**	/	*59*
Waist-to-height ratio	**0.53**	/	*0*.*11*	**0.57**	/	*0*.*12*
*Waist-to-height ratio ≥ 0*.*5*	**65.9%**	/	*4312*	**82.7%**	/	*115*
Smoking Status	** **			** **		* *
*Never smoker*	**51.4%**	/	*3365*	**48.2%**	/	*67*
*Former smoker*	**23.6%**	/	*1545*	**32.4%**	/	*45*
*Current smoker*	**25.0%**	/	*1637*	**19.4%**	/	*27*
Alcohol consumption, g/d	**3.6**	/	*10*.*2*	**2.8**	/	*10*.*5*
*At-risk alcohol consumption*^*†*^	**8.2%**	/	*534*	**6.5%**	/	*9*
Health insurance type	** **			** **		* *
*Statutory*	**92.3%**	/	*6045*	**97.8%**	/	*136*
*Private*	**6.3%**	/	*413*	**2.6%**	/	*3*
*Other*	**1.4%**	/	*89*	**0.0%**	/	*0*
Number of chronic diseases^‡^	** **			** **		* *
*None*	**27.7%**	/	*1810*	**7.2%**	/	*10*
*One*	**47.4%**	/	*3100*	**36.0%**	/	*50*
*Two*	**17.8%**	/	*1163*	**31.7%**	/	*44*
*Three or more*	**7.2%**	/	*474*	**25.2%**	/	*35*
Currently taking medication^§^	**68.4%**	/	*4478*	**86.3%**	/	*120*
Number of current medications^§^	**2.0**	/	*4*.*0*	**5.0**	/	*6*.*0*

Median / IQR or Proportion / N

* Data was pooled from SHIP-2 and SHIP Trend-0 cohorts for analyses.

^†^ Women: ≥ 20 g/d; Men: ≥ 30 g/d

^‡^ hypertension, myocardial infarction, stroke, diabetes, cancer, pulmonary / kidney / liver disease

^§^ Excluding contraceptives

### Outpatient healthcare utilization

Tables 2 and 3 show descriptive statistics and results from regression models regarding outpatient consultations by ICU treatment status. In the unadjusted data, post-ICU subjects showed higher utilization of almost all outpatient services. Regarding the previous year, 98.6% of post-ICU subjects reported any outpatient consultation, with an average of 11.2 visits and total costs of 373.1 €. In comparison, 89.2% of those without ICU treatment reported any consultation, with an average of 6.5 visits and total costs of 176.3 €. Regarding consultations within the previous four weeks, this effect was more pronounced: 76% of post-ICU subjects reported any consultation and 1.8 visits on average, compared to 44% of subjects without ICU treatment who had 1.5 visits on average. Post-ICU subjects more frequently reported taking any medication (86.3% vs. 68.4%) with more medications on average (4.7 vs. 2.7). In adjusted regression models, ICU treatment was associated with a higher probability (PR 1.05 [CI: 1.03; 1.07]), number (PC +58.0% [CI: 22.8; 103.2]) and costs (PC +64.1% [CI: 32.0; 103.9]) of outpatient consultations in the previous year. This observation was more pronounced for consultations in the previous 4 weeks (probability: PR 1.32 [CI: 1.21; 1.45], number: PC +73.6% [CI: 33.3; 126.2]). ICU treatment was also associated with more specialist consultations (probability: PR 1.13 [CI: 1.09; 1.16], number: PC +65.4% [CI: 23.6; 121.3]) and higher costs (PC +73.3% [CI: 17.8; 155.1]), specifically internal medicine (PR 1.67 [CI: 1.45; 1.92]), surgery (PR 2.42 [CI: 1.92; 3.05]), psychiatry (PR 2.25 [CI: 1.30; 3.90]), and orthopedics (PR 1.54 [CI: 1.11; 2.14]). For psychiatry and orthopedics, only the probability of consultations was higher, but not the number or associated costs. There was no significant effect regarding general practitioner consultations. ICU treatment was also associated with a higher probability of taking any medication (PR 1.08 [CI: 1.02; 1.14]) and a higher number of medications (PC +37.8% [CI: 17.7; 61.5]).

**Table 2:** Outpatient consultations and associated costs in the previous 12 months by ICU treatment status.

	Self-reported ICU treatment in previous 12 months								
	Descriptive statistics							Adjusted regression models^‡^	
	No (97.9%)			Yes (2.1%)				* *	
	(N = 6,547)			(N = 139)					
Variable	**Proportion** / *N* or **Geometric mean** / *Geom*. *SD*							Prevalence ratio (PR) [95% CI]^§^ or Percent change (Δ) [95% CI]^‖^^,^^¶^	
Any consultation (12 months)	**89.2%**	/	*5842*	**98.6%**	/	*137*	PR	**1.05**	[1.03; 1.07]
Total number of consultations*	**6.46**	/	*2*.*36*	**11.19**	/	*1*.*98*	Δ	**+ 58.0%**	[+ 22.8%; + 103.2%]
Total consultation costs, €*	**176.30**	/	*2*.*71*	**373.11**	/	*2*.*19*	Δ	**+ 64.1%**	[+ 32.0%; + 103.9%]
Any consultation (4 weeks)	**43.8%**	/	*2866*	**76.3%**	/	*106*	PR	**1.32**	[1.21; 1.45]
Number of consultations	**1.47**	/	*1*.*69*	**1.80**	/	*1*.*83*	Δ	**+ 73.6%**	[+ 33.3%; + 126.2%]
Currently taking medication^†^	**68.4%**	/	*4478*	**86.3%**	/	*120*	PR	**1.08**	[1.02; 1.14]
Number of medications^†^	**2.68**	/	*2*.*10*	**4.67**	/	*2*.*04*	Δ	**+ 37.8%**	[+ 17.7%; + 61.5%]
General practitioner	**76.1%**	/	*4980*	**79.1%**	/	*110*	PR	0.90	[0.74; 1.09]
Number of consultations*	**2.93**	/	*2*.*19*	**4.73**	/	*1*.*95*	Δ	- 7.4%	[- 49.6%; + 69.9%]
Consultation costs, €*	**55.17**	/	*2*.*19*	**89.12**	/	*1*.*95*	Δ	- 8.3%	[- 52.7%; + 77.5%]
Any specialist consultation	**77.3%**	/	*5062*	**96.4%**	/	*134*	PR	**1.13**	[1.09; 1.16]
Number of consultations*	**4.25**	/	*2*.*47*	**6.26**	/	*2*.*57*	Δ	**+ 65.4%**	[+ 23.6%; +121.3%]
Consultation costs, €*	**143.35**	/	*2*.*80*	**257.04**	/	*2*.*90*	Δ	**+ 73.3%**	[+ 17.8%; + 155.1%]

* Number and costs of consultations: SHIP-2 only (N = 2,324)

^†^ Excluding contraceptives

^‡^ Adjusted for age, gender, number of chronic diseases, cohort (SHIP-2/Trend-0), with balancing weights

^§^ Any consultation or medication intake: Poisson regression

^‖^Number of consultations or medications: Negative binomial regression

^¶^Consultation costs: Generalized linear models with gamma-distribution and log-link function

**Table 3:** Specialist consultations and associated costs in the previous 12 months by ICU treatment status.

	Self-reported ICU treatment in previous 12 months								
	Descriptive statistics							Adjusted regression models^†^	
	No (97.9%)			Yes (2.1%)				* *	
	(N = 6,547)			(N = 139)					
Variable	**Proportion** / *N* or **Geometric mean** / *Geom*. *SD*							Prevalence ratio (PR) [95% CI]^‡^ or Percent change (Δ) [95% CI]^§^^,^^‖^	
Internal medicine	**28.2%**	/	*1846*	**64.8%**	/	*90*	PR	**1.67**	[1.45; 1.92]
Number of consultations*	**2.30**	/	*2*.*14*	**3.37**	/	*2*.*34*	Δ	**+ 85.0%**	[+ 30.8%; + 161.6%]
Consultation costs, €^a^	**140.73**	/	*2*.*14*	**206.12**	/	*2*.*33*	Δ	**+ 88.3%**	[+ 31.8%; + 168.9%]
Surgery	**16.8%**	/	*1097*	**43.2%**	/	*60*	PR	**2.42**	[1.92; 3.05]
Number of consultations*	**1.91**	/	*1*.*99*	**2.09**	/	*2*.*24*	Δ	**+ 134.5%**	[+ 45.9%; + 276.8%]
Consultation costs, €*	**77.51**	/	*2*.*00*	**85.63**	/	*2*.*25*	Δ	**+ 133.2%**	[+ 40.5%; + 286.8%]
Neurology	**9.7%**	/	*634*	**22.3%**	/	*31*	PR	1.20	[0.79; 1.82]
Number of consultations*	**2.12**	/	*2*.*12*	**1.74**	/	*1*.*84*	Δ	- 53.1%	[- 81.5%; + 18.7%]
Consultation costs, €*	**88.76**	/	*2*.*12*	**72.52**	/	*1*.*84*	Δ	- 58.9%	[- 89.1%; + 55.5%]
Psychiatry or Psychotherapy	**5.0%**	/	*324*	**13.7%**	/	*19*	PR	**2.25**	[1.30; 3.90]
Number of consultations*	**4.79**	/	*2*.*89*	**4.20**	/	*2*.*40*	Δ	+ 95.5%	[- 30.3%; + 448.6%]
Consultation costs, €*	**351.27**	/	*2*.*89*	**308.12**	/	*2*.*40*	Δ	+ 107.2%	[- 28.6%; + 501.2%]
Dermatology	**18.5%**	/	*1208*	**22.3%**	/	*31*	PR	0.98	[0.66; 1.45]
Number of consultations*	**1.63**	/	*1*.*81*	**1.58**	/	*1*.*72*	Δ	-24.7%	[- 66.9%; + 71.4%]
Consultation costs, €*	**28.80**	/	*1*.*81*	**28.03**	/	*1*.*71*	Δ	- 23.8%	[- 68.4%; + 83.6%]
Ophthalmology	**30.2%**	/	*1974*	**40.3%**	/	*56*	PR	1.09	[0.89; 1.33]
Number of consultations*	**1.52**	/	*1*.*81*	**1.58**	/	*2*.*20*	Δ	+ 58.3%	[- 12.0%; + 184.8%]
Consultation costs, €*	**49.44**	/	*1*.*81*	**51.43**	/	*2*.*20*	Δ	+ 6.6%	[- 42.2%; + 96.6%]
Otorhinolaryngology	**16.5%**	/	*1081*	**19.4%**	/	*27*	PR	1.37	[0.93; 2.02]
Number of consultations*	**1.54**	/	*1*.*80*	**1.67**	/	*1*.*99*	Δ	- 3.8%	[- 44.0%; + 65.3%]
Consultation costs, €*	**38.10**	/	*1*.*80*	**40.83**	/	*1*.*99*	Δ	- 37.9%	[- 71.2%; + 33.9%]
Orthopedics	**18.5%**	/	*1214*	**28.8%**	/	*40*	PR	**1.54**	[1.11; 2.14]
Number of consultations*	**1.93**	/	*1*.*92*	**2.15**	/	*2*.*22*	Δ	+ 81.6%	[- 7.9%; 258.3%]
Consultation costs, €*	**46.03**	/	*1*.*92*	**51.01**	/	*2*.*21*	Δ	+ 71.9%	[- 16.1%; + 252.4%]

* Number and costs of consultations: SHIP-2 only (N = 2,324)

^†^ Adjusted for age, gender, number of chronic diseases, cohort (SHIP-2/Trend-0), with balancing weights

^‡^ Any consultation: Poisson regression

^§^ Number of consultations: Negative binomial regression

^‖^ Consultation costs: Generalized linear models with gamma-distribution and log-link function

Omitted: Urology, Gynecology (Table B in S1 Appendix)

### Health-related quality of life

Table 4 shows results for quality of life analyses. In the unadjusted data, post-ICU subjects more frequently reported impairments in all five EQ-5D subdomains, and accordingly showed lower health-related quality of life (EQ-5D index value 0.77 vs. 0.88). In adjusted regression models, the effect of post-ICU status on the EQ-5D index value was Δ -13.7% [CI: -27.0; -0.3], with a significantly higher probability of impairments in the domains self-care (PR 3.41 [CI: 1.71; 6.82]) and usual activity (PR 1.68 [CI: 1.21; 2.34]).

**Table 4:** EQ-5D-3L index value and subdomains by ICU treatment status.

	ICU treatment in previous 12 months								
	Descriptive statistics							Adjusted regression models^†^	
	No (97.9%)			Yes (2.1%)				* *	
	(N = 6,547)*			(N = 139)					
Variable	**Geometric mean** / Geom. SD or **Proportion** / *N*							Percent change (Δ) [95% CI]^‡^ or Prevalence ratio (PR) [95% CI]^§^	
EQ5D-3L index value	**0.88**	/	*1*.*29*	**0.77**	/	*1*.*57*	Δ	**- 13.7%**	[- 27.0%; - 0.3%]
*Any impairment in EQ-5D-3L Subdomains*				** **		* *	* *	** **	
Mobility	**13.0%**	/	*850*	**26.6%**	/	*37*	PR	1.27	[0.93; 1.71]
Self-Care	**1.6%**	/	*105*	**6.5%**	/	*9*	PR	**3.41**	[1.71; 6.82]
Usual Activity	**10.9%**	/	*714*	**23.0%**	/	*32*	PR	**1.68**	[1.21; 2.34]
Pain/Discomfort	**55.3%**	/	*3608*	**70.5%**	/	*98*	PR	1.10	[0.99; 1.21]
Anxiety/Depression	**18.7%**	/	*1218*	**23.0%**	/	*32*	PR	1.09	[0.74; 1.59]

* N = 18 observations (< 1%) excluded (EQ-5D not available)

^†^ Adjusted for age, gender, number of chronic diseases, cohort (SHIP-2/Trend-0), with balancing weights

^‡^ EQ-5D index value: fractional response model with average marginal effects

^§^ EQ-5D subdomain impairments: Poisson regression

**Fig 1:**
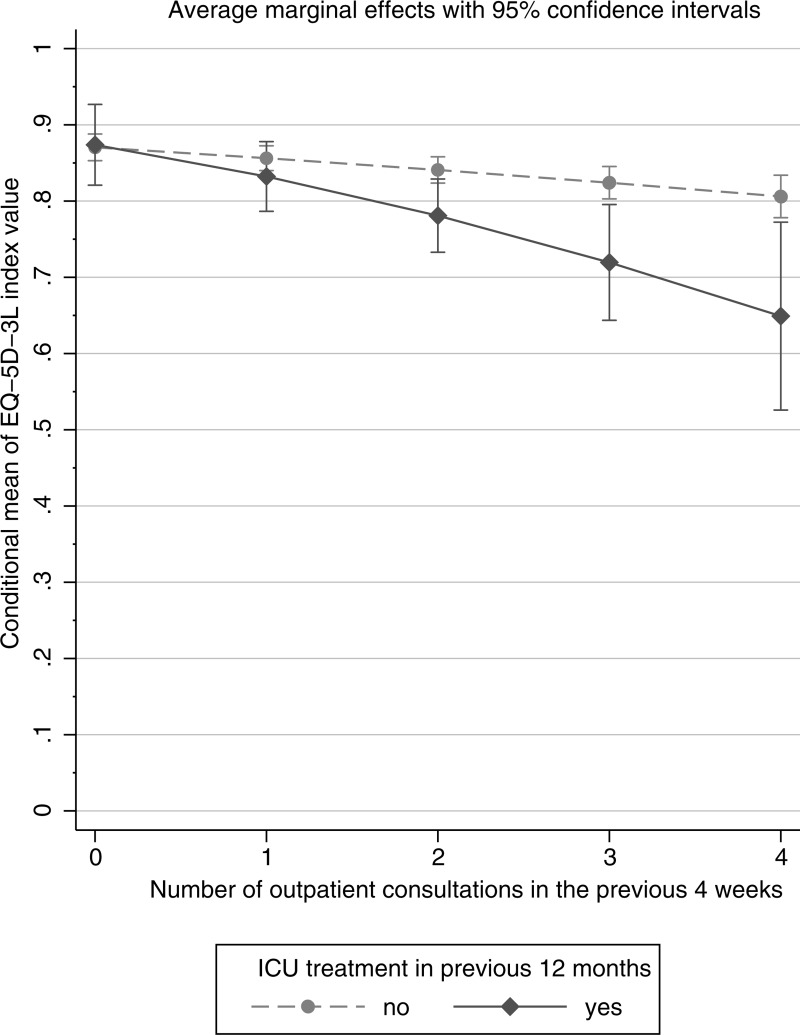
Association of the EQ-5D-3L index value with outpatient consultations in the previous 4 weeks by ICU treatment status (average marginal effects from a fractional response model).

Fig 1 shows the association of health-related quality of life with medical consultations and post-ICU status. The number of consultations in the previous four weeks was inversely associated with the EQ-5D index value, and this effect was more pronounced in post-ICU subjects.

### Sensitivity analyses

In regression analyses, we calculated unadjusted and fully adjusted models for comparison, and additionally calculated E-values to estimate the potential impact of unmeasured confounding (Tables B and C in S1 Appendix). [38] For example, an unmeasured confounder would have to increase the probability of a surgical consultation by 4.27-fold beyond the measured confounders to fully explain away the PR estimate for ICU treatment of 2.42, and by 3.25-fold to bring its lower confidence limit below 1.0, respectively.

## Discussion

In this study, we investigated the association of ICU treatment with outpatient health services utilization and quality of life. In summary, we were able to show that ICU treatment is associated with an increased probability of outpatient specialist consultations, specifically internal medicine, surgery, psychiatry, and orthopedics, but not general practitioner consultations. ICU treatment was also associated with an increased number of outpatient consultations and related costs. In addition, ICU treatment was associated with a higher probability of taking any medication as well as a higher number of medications. We also found that ICU treatment is associated with a 13.7% reduction of health-related quality of life (EQ-5D index value) and a higher probability of impairments in self-care and usual activities within the first year following critical illness. Quality of life was also inversely associated with the number of outpatient consultations.

In this cross-sectional analysis of population-based data, we found a prevalence of ICU treatment in the previous year of 2.1% among participants, which is congruent with official statistical data from Germany: In 2012, at a total German population of 80,523,746, there were 2,127,037 ICU treatment cases, which results in a prevalence of 2.64%. [42, 43] At an estimated one-year mortality of about 20%, this results in a hypothetical prevalence of survivors at one year post-ICU of 2.11%, which validates our findings. [44, 45] While dedicated critical care cohort studies may feature larger numbers of post-ICU subjects, the strength of this study consequently lies in the fact that it uses representative population-based data and compares post-ICU resource utilization to that of the general population.

Previous research has shown that critical illness and ICU treatment is associated with an increase in healthcare resource utilization and costs, mostly attributable to hospital readmission. [10–17, 46] The majority of these studies are based on ICU or hospital cohorts and are thus not comparable to our study that relied on a sample of the general population. One previous study of a cohort of ARDS survivors reported results on outpatient specialist visits and found that internal medicine and psychiatry were among the most frequently reported consultations following intensive care, which is consistent with our findings. [17] Another recently published study of a cohort of ARDS survivors from Germany reported detailed results on resource utilization with overall comparable numbers for outpatient visits, with the most notable deviations being more general practitioner and fewer surgeon visits. [18] One study of critically ill older patients with a matched control group also reported more general practitioner consultations and higher medication intake for post-ICU subjects. [47] In contrast, another cohort study of post-ICU patients found no change in the number of general practitioner consultations or medications in the majority of the participants. [48]

An interesting finding from our study is that ICU treatment is associated with more specialist, but not general practitioner consultations. It is unclear why general practitioners were not more frequently consulted following ICU treatment, but a possible explanation is the free choice of treatment providers including specialists in the German healthcare system. Further qualitative studies might elucidate these patients’ motivation to directly consult a specialist instead of a general practitioner. The finding that surgeons and orthopedists are more likely to be consulted can be explained by postoperative ICU stays and surgical follow-up, including orthopedists in case of orthopedic surgery. Similarly, the higher probability and number of internal medicine consultations, as well as the increased medication intake, can be explained by medical ICU stays related to organ dysfunction such as sepsis or cardiovascular events. Our results indicate that patients are more likely to consult a psychiatrist following ICU treatment, which might be explained by neuropsychiatric sequelae, but do not receive a substantially different psychiatric treatment in terms of the number of therapy sessions.

Short- and long-term impairments in quality of life in survivors of critical illness have previously been demonstrated. [6, 7] Our analyses of the EQ-5D instrument showed a 13.7% reduction of health-related quality of life (EQ-5D index value) and a higher probability of impairments in self-care and usual activities, which confirms previous findings. [16, 49, 50] As a novel result, we additionally found that the quality of life measure was inversely associated with the number of outpatient consultations in the previous four weeks, significantly more so for post-ICU subjects (Fig 1). Our results indicate that low quality of life is associated with frequent specialist consultations for this subgroup of patients.

ICU treatment is associated with continuation of inappropriate medication after discharge, as well as discontinuation of maintenance medication for chronic diseases, possibly resulting in increased morbidity and mortality. [51, 52] The Society of Critical Care Medicine has recommended integration of a pharmacist into ICU teams, and the benefits of this involvement have previously been demonstrated. [53–55] A recent study investigated the utility of critical care pharmacist visits in an ICU recovery center with promising results. [56] In our study, ICU treatment was associated with a 38% increase of the number of medications within the following year, supporting the idea that these patients might also benefit from clinical pharmacist visits in the follow-up period.

We acknowledge some limitations of our study. First of all, the temporal association of comorbidities, ICU treatment, and outpatient consultations, all reported for the year prior to the respective examination, cannot be determined more exactly due to the cross-sectional study design. However, we have implemented comprehensive adjustments into our analyses to address these uncertainties.

Second, since SHIP is a general population-based cohort study and not a dedicated critical care cohort study, detailed data on ICU diagnoses and treatment modalities are not available. Using a population-based cohort for the research question at hand offers some unique advantages, however, mostly through comparison to the general population as described above. While reported ICU treatment was the exposure variable for our analyses, it is important to note that it also indicates critical illness. Accordingly, we cannot determine the cause and severity of critical illness or the intensity of ICU treatment, which is typically classified using the sequential organ failure assessment score (SOFA) or a comparable system. [57] We have addressed this uncertainty by adjusting for morbidity using the number of present chronic conditions, under the assumption that multimorbid patients required more intensive treatment. In sensitivity analyses using E-values, we found that substantial confounding would be needed to explain most of the effect estimates with significant results. However, we cannot fully exclude residual confounding due to premorbid disease burden including psychiatric disease.

Another limitation comes from the fact that healthcare services use was self-reported and could not be validated. However, self-reports of outpatient consultations and hospital admissions are highly correlated with actual use of services, and greater utilization of healthcare services is typically associated with underreporting, so our study most likely provides conservative estimates. [58, 59] Compared to representative data for the use of medical services in Germany, we found good overall agreement, especially regarding the group without ICU treatment, which further validates our results. [60]

## Conclusions

ICU treatment is associated with an increased utilization of outpatient specialist services, higher medication intake, and impaired quality of life. Furthermore, quality of life is inversely associated with the frequency of outpatient consultations. Further research into post-ICU follow-up care is needed to develop treatment strategies that are effective for improving quality of life and reducing healthcare costs. It has been proposed that future trials should focus on multi-disciplinary follow-up strategies, which might include physicians as well as other professions such as nurses, physiotherapists, occupational, speech and language therapists, psychologists, dieticians, social workers or clinical pharmacists. [56, 61, 62] Our study contributes to this goal by identifying specific medical disciplines that should be considered for multi-disciplinary post-ICU interventions.

## Supporting information

S1 Appendix Supplementary material. Table A. Entropy balancing diagnostics. Table B. Outpatient consultations and associated costs in the previous 12 months by ICU treatment status, including sensitivity analyses. Table C. EQ-5D-3L index value and subdomains by ICU treatment status, including sensitivity analyses.
(PDF)
