# Supplementary material for: The association of intensive care with utilization and costs of outpatient healthcare services and quality of life
Source: PLoS One. 2019 Sep 20;14(9):e0222671. doi: 10.1371/journal.pone.0222671 (PMC6754134; doi:10.1371/journal.pone.0222671)
Supplement: S1 Appendix — Table A. Entropy balancing diagnostics. Table B. Outpatient consultations and associated costs in the previous 12 months by ICU treatment status, including sensitivity analyses. Table C. EQ-5D-3L index value and subdomains by ICU treatment status, including sensitivity analyses. (PDF) [file pone.0222671.s001.pdf]

S1 Appendix: Supplementary Material

Table A: Entropy balancing diagnostics

	ICU treatment in previous 12 months						Standardized Differences
	No (97.9%) (N=6,547)			Yes (2.1%) (N=139)			
Variable	Mean	Variance	Skewness	Mean	Variance	Skewness	Pre
Age	54.42	239.3	-0.100	61.39	176.2	-0.461	0.52498084
Gender	0.480	0.250	0.080	0.698	0.212	-0.861	0.47263432
Age-gender interaction term	26.43	875.2	0.447	43.70	928.0	-0.560	0.56707728
Body mass index	28.25	26.41	0.817	30.76	41.26	0.312	0.39050868
Waist-to-height ratio	0.544	0.007	0.469	0.593	0.010	0.124	0.49411267
Education (school years)	12.07	6.592	-0.075	11.75	7.607	-0.317	-0.11541875
Equivalent household income	1336	454704	1.269	1197	320446	1.666	-0.24579014
Alcohol (g/d)	8.607	182.5	3.505	6.395	109.9	2.313	-0.21106285
Relationship status	0.759	0.183	-1.214	0.810	0.155	-1.580	0.12855333
Insurance type	1.045	0.069	1.983	1.017	0.016	7.564	-0.22221085
Smoking status	0.747	0.699	0.502	0.625	0.581	0.745	-0.15993731
Physical inactivity	0.320	0.218	0.771	0.373	0.236	0.525	0.10877801
Diabetes	0.122	0.107	2.313	0.343	0.227	0.662	0.46419796
Hypertension	0.654	0.226	-0.648	0.843	0.133	-1.885	0.51690632
Myocardial infarction	0.026	0.025	5.976	0.084	0.078	2.997	0.20915668
Stroke	0.018	0.018	7.223	0.141	0.122	2.067	0.35119328
Lung disease	0.098	0.089	2.697	0.097	0.088	2.718	-0.00369515
Kidney disease	0.034	0.033	5.133	0.166	0.139	1.799	0.35245848
Liver disease	0.082	0.075	3.058	0.235	0.181	1.252	0.36016911
Cancer	0.069	0.065	3.390	0.164	0.138	1.814	0.25493437
	Reweighted						Post
Age	61.39	176.2	-0.461	61.39	176.2	-0.461	0.00000138
Gender	0.698	0.211	-0.861	0.698	0.212	-0.861	-0.00000294
Age-gender interaction term	43.70	928.0	-0.560	43.70	928.0	-0.560	-0.00000206
Body mass index	30.76	41.26	0.312	30.76	41.26	0.312	-0.00000002
Waist-to-height ratio	0.593	0.010	0.124	0.593	0.010	0.124	-0.00000027
Education (school years)	11.75	7.607	-0.317	11.75	7.607	-0.317	-0.00000096
Equivalent household income	1197	320447	1.666	1197	320446	1.666	-0.00000083
Alcohol (g/d)	6.40	109.9	2.313	6.395	109.9	2.313	-0.00002295
Relationship status	0.810	0.154	-1.580	0.810	0.155	-1.580	-0.00000069
Insurance type	1.017	0.033	2.457	1.017	0.016	7.564	-0.00000122
Smoking status	0.625	0.577	0.745	0.625	0.581	0.745	-0.00000205
Physical inactivity	0.373	0.234	0.525	0.373	0.236	0.525	-0.00000123
Diabetes	0.343	0.225	0.662	0.343	0.227	0.662	0.00000085
Hypertension	0.843	0.132	-1.885	0.843	0.133	-1.885	-0.00000113
Myocardial infarction	0.084	0.077	2.997	0.084	0.078	2.997	0.00000115
Stroke	0.141	0.121	2.067	0.141	0.122	2.067	0.00000190
Lung disease	0.097	0.088	2.718	0.097	0.088	2.718	-0.00000030
Kidney disease	0.166	0.138	1.799	0.166	0.139	1.799	0.00000013
Liver disease	0.235	0.180	1.252	0.235	0.181	1.252	-0.00000127
Cancer	0.164	0.137	1.814	0.164	0.138	1.814	0.00000040

Comparison of covariates before and after reweighting by entropy balancing (ebalance for Stata, Hainmueller & Xu 2013), with applied stabilized base weigths from inverse probability weighting for drop-out adjustment

S1 Appendix: Supplementary Material

Table B: Outpatient consultations and associated costs in the previous 12 months by ICU treatment status, including sensitivity analyses

	Self-reported ICU treatment in previous 12 months					Sensitivity analysis			
	Descriptive statistics			Regression models		E-value[#]			
	No (97.9%) (N=6,547)	Yes (2.1%) (N=139)		Effect on probability of visit: Prevalence ratio (PR) [95% CI][§] Effect on number of visits and costs: % change (Δ) [95% CI][‖,¶]		E(PR)	E(CI)	E(PR)	E(CI)
Variable	**Proportion** / *N* or **Geometric mean** / *Geom. SD*			**Adjusted**[‡]	Unadjusted	**Adjusted**[‡]		Unadjusted	
Any consultation (12 months)	**89.2%** / *5842*	**98.6%** / *137*	PR	**1.05** [1.03; 1.07]	1.10 [1.08; 1.13]	1.27	1.19	1.44	1.38
Total number of consultations[*]	**6.46** / *2.36*	**11.19** / *1.98*	Δ	**+58.0%** [+22.8%; +103.2%]	+71.6% [+32.9%; +121.6%]	2.54	1.76	2.82	1.99
Total consultation costs, €[*]	**176.30** / *2.71*	**373.11** / *2.19*	Δ	**+64.1%** [+32.0%; +103.9%]	+96.2% [+36.4%; +182.2%]	2.67	1.97	3.34	2.07
Any consultation (4 weeks)	**43.8%** / *2866*	**76.3%** / *106*	PR	**1.32** [1.21; 1.45]	1.74 [1.58; 1.92]	1.97	1.70	2.88	2.54
Number of consultations	**1.47** / *1.69*	**1.80** / *1.83*	Δ	**+73.6%** [+33.3%; +126.2%]	+120.9% [+78.4%; +173.7%]	2.87	2.00	3.84	2.97
Currently taking medication[†]	**68.4%** / *4478*	**86.3%** / *120*	PR	**1.08** [1.02; 1.14]	1.26 [1.18; 1.35]	1.36	1.15	1.84	1.64
Number of medications[†]	**2.68** / *2.10*	**4.67** / *2.04*	Δ	**+37.8%** [+17.7%; +61.5%]	+107.3% [+72.1%; +149.7%]	2.10	1.63	3.57	2.84
General practitioner	**76.1%** / *4980*	**79.1%** / *110*	PR	0.90 [0.74; 1.09]	1.04 [0.95; 1.13]	1.48	1.00	1.25	1.00
Number of consultations[*]	**2.93** / *2.19*	**4.73** / *1.95*	Δ	-7.4% [-49.6%; +69.9%]	+49.1% [+10.4%; +101.3%]	1.38	1.00	2.35	1.44
Consultation costs, €[*]	**55.17** / *2.19*	**89.12** / *1.95*	Δ	-8.3% [-52.7%; +77.5%]	+48.9% [+6.6%; +107.8%]	1.41	1.00	2.34	1.33
Any specialist consultation	**77.3%** / *5062*	**96.4%** / *134*	PR	**1.13** [1.09; 1.16]	1.25 [1.20; 1.29]	1.50	1.40	1.80	1.70
Number of consultations[*]	**4.25** / *2.47*	**6.26** / *2.57*	Δ	**+65.4%** [+23.6%; +121.3%]	+85.1% [+35.1%; +153.6%]	2.69	1.78	3.11	2.04
Consultation costs, €[*]	**143.35** / *2.80*	**257.04** / *2.90*	Δ	**+73.3%** [+17.8%; +155.1%]	+121.9% [+32.9%; +270.4%]	2.86	1.63	3.86	1.99
Specialist consultations by type [*]									
Internal medicine	**28.2%** / *1846*	**64.8%** / *90*	PR	**1.67** [1.45; 1.92]	2.30 [2.02; 2.61]	2.73	2.26	4.02	3.45
Number of consultations	**2.30** / *2.14*	**3.37** / *2.34*	Δ	**+85.0%** [+30.8%; +161.6%]	+220.8% [+75.8%; +485.5%]	3.10	1.94	5.87	2.91
Consultation costs, €	**140.73** / *2.14*	**206.12** / *2.33*	Δ	**+88.3%** [+31.8%; +168.9%]	+219.7% [+67.3%; +510.8%]	3.17	1.97	5.85	2.73
Surgery	**16.8%** / *1097*	**43.2%** / *60*	PR	**2.42** [1.92; 3.05]	2.58 [2.11; 3.14]	4.27	3.25	4.59	3.65
Number of consultations	**1.91** / *1.99*	**2.09** / *2.24*	Δ	**+134.5%** [+45.9%; +276.8%]	+171.7% [+23.5%; +498.0%]	4.12	2.28	4.88	1.77
Consultation costs, €	**77.51** / *2.00*	**85.63** / *2.25*	Δ	**+133.2%** [+40.5%; +286.8%]	+173.9% [+13.5%; +561.2%]	4.09	2.16	4.92	1.53
Neurology	**9.7%** / *634*	**22.3%** / *31*	PR	1.20 [0.79; 1.82]	2.30 [1.67; 3.17]	1.69	1.00	4.04	2.74
Number of consultations	**2.12** / *2.12*	**1.74** / *1.84*	Δ	-53.1% [-81.5%; +18.7%]	+49.4% [-54.3%; +388.6%]	3.69	1.00	2.35	1.00
Consultation costs, €	**88.76** / *2.12*	**72.52** / *1.84*	Δ	-58.9% [-89.1%; +55.5%]	+49.2% [-57.3%; +420.9%]	4.30	1.00	2.35	1.00
Psychiatry or Psychotherapy	**5.0%** / *324*	**13.7%** / *19*	PR	**2.25** [1.30; 3.90]	2.76 [1.79; 4.25]	3.93	1.93	4.97	2.99
Number of consultations	**4.79** / *2.89*	**4.20** / *2.40*	Δ	+95.5% [-30.3%; +448.6%]	+90.4% [-80.4%; +1752%]	3.32	1.00	3.22	1.00
Consultation costs, €	**351.27** / *2.89*	**308.12** / *2.40*	Δ	+107.2% [-28.6%; +501.2%]	+91.0% [-74.8%; +1346%]	3.56	1.00	3.23	1.00
Dermatology	**18.5%** / *1208*	**22.3%** / *31*	PR	0.98 [0.66; 1.45]	1.21 [0.88; 1.66]	1.19	1.00	1.71	1.00
Number of consultations	**1.63** / *1.81*	**1.58** / *1.72*	Δ	-24.7% [-66.9%; +71.4%]	+7.0% [-48.7%; +123.2%]	1.99	1.00	1.34	1.00
Consultation costs, €	**28.80** / *1.81*	**28.03** / *1.71*	Δ	-23.8% [-68.4%; +83.6%]	+7.2% [-48.4%; +122.6%]	1.95	1.00	1.35	1.00
Ophthalmology	**30.2%** / *1974*	**40.3%** / *56*	PR	1.09 [0.89; 1.33]	1.34 [1.09; 1.64]	1.41	1.00	2.01	1.40
Number of consultations	**1.52** / *1.81*	**1.58** / *2.20*	Δ	+58.3% [-12.0%; +184.8%]	+38.9% [-13.7%; +123.7%]	2.54	1.00	2.12	1.00
Consultation costs, €	**49.44** / *1.81*	**51.43** / *2.20*	Δ	+6.6% [-42.2%; +96.6%]	+39.0% [-19.8%; +141.0%]	1.33	1.00	2.13	1.00
Otorhinolaryngology	**16.5%** / *1081*	**19.4%** / *27*	PR	1.37 [0.93; 2.02]	1.18 [0.83; 1.66]	2.09	1.00	1.63	1.00
Number of consultations	**1.54** / *1.80*	**1.67** / *1.99*	Δ	-3.8% [-44.0%; +65.3%]	+6.8% [-50.8%; +131.6%]	1.24	1.00	1.34	1.00
Consultation costs, €	**38.10** / *1.80*	**40.83** / *1.99*	Δ	-37.9% [-71.2%; +33.9%]	+5.6% [-53.4%; +139.5%]	2.60	1.00	1.30	1.00
Orthopedics	**18.5%** / *1214*	**28.8%** / *40*	PR	**1.54** [1.11; 2.14]	1.55 [1.19; 2.03]	2.45	1.45	2.48	1.66
Number of consultations	**1.93** / *1.92*	**2.15** / *2.22*	Δ	+81.6% [-7.9%; 258.3%]	+54.9% [-26.3%; +225.3%]	3.03	1.00	2.47	1.00
Consultation costs, €	**46.03** / *1.92*	**51.01** / *2.21*	Δ	+71.9% [-16.1%; +252.4%]	+54.0% [-28.9%; +233.5%]	2.83	1.00	2.45	1.00
Urology	**19.7%** / *1289*	**33.1%** / *46*	PR	1.03 [0.79; 1.34]	1.68 [1.32; 2.14]	1.19	1.00	2.75	1.97
Number of consultations	**1.59** / *1.78*	**2.27** / *2.47*	Δ	+28.1% [-36.4%; +158.0%]	+121.8% [+23.0%; +299.9%]	1.88	1.00	3.86	1.76
Consultation costs, €	**36.80** / *1.78*	**52.43** / *2.47*	Δ	-53.4% [-81.6%; +18.0%]	+121.2% [+15.5%; +323.8%]	3.72	1.00	3.85	1.58
Gynecology (females only)	**61.6%** / *2115*	**55.6%** / *25*	PR	0.88 [0.66; 1.17]	0.90 [0.69; 1.17]	1.52	1.00	1.46	1.00
Number of consultations	**1.28** / *1.59*	**1.76** / *1.86*	Δ	-2.4% [-49.1%; +87.4%]	+17.7% [-29.8%; +97.2%]	1.18	1.00	1.63	1.00
Consultation costs, €	**36.07** / *1.59*	**49.98** / *1.89*	Δ	-31.0% [-72.9%; +76.2%]	+19.7% [-35.6%; +122.4%]	2.25	1.00	1.68	1.00

[*]Number and costs of consultations: SHIP-2 only (N=2,324)

[†]Excluding contraceptives

[‡]Adjusted for age, gender, number of chronic diseases, cohort (SHIP-2/Trend-0), with balancing weights

[§]Any consultation or medication intake: Poisson regression with robust standard errors

[‖]Number of consultations or medications: Negative binomial regression

[¶]Consultation costs: Generalized linear models with gamma-distribution and a log-link function

[#] VanderWeele & Ding, Ann Int Med 2017

Table C: EQ-5D-3L index values and subdomains by ICU treatment status, including sensitivity analyses

	ICU treatment in previous 12 months					Sensitivity analysis			
	Descriptive statistics			Regression models		E-value[‖]			
	No (97.9%) (N=6,547)[*]	Yes (2.1%) (N=139)		Percent change (Δ) [95% CI][‡] or Prevalence ratio (PR) [95% CI][§]		E(PR)	E(CI)	E(PR)	E(CI)
Variable	**Geometric mean** / Geom. SD or **Proportion** / *N*			**Adjusted**[†]	Unadjusted	**Adjusted**[†]		Unadjusted	
EQ5D-3L index value	**0.88** / *1.29*	**0.77** / *1.57*	Δ	**-13.7%** [-27.0%; -0.3%]	-8.5% [-12.9%; -4.1%]	n/a		n/a	
Any impairment in EQ-5D-3L Subdomains									
Mobility	**13.0%** / *850*	**26.6%** / *37*	RR	1.27 [0.93; 1.71]	2.04 [1.54; 2,71]	1.85	1.00	3.51	2.45
Self-Care	**1.6%** / *105*	**6.5%** / *9*	RR	**3.41** [1.71; 6.82]	4.03 [2.08; 7.79]	6.29	2.81	7.52	3.58
Usual Activity	**10.9%** / *714*	**23.0%** / *32*	RR	**1.68** [1.21; 2.34]	2.11 [1.54; 2.88]	2.75	1.71	3.63	2.45
Pain/Discomfort	**55.3%** / *3608*	**70.5%** / *98*	RR	1.10 [0.99; 1.21]	1.28 [1.14; 1.42]	1.44	1.00	1.87	1.55
Anxiety/Depression	**18.7%** / *1218*	**23.0%** / *32*	RR	1.09 [0.74; 1.59]	1.23 [0.91; 1.68]	1.39	1.00	1.77	1.00

[*]N=18 observations (< 1%) excluded (EQ-5D not available)

[†]Adjusted for age, gender, number of chronic diseases, cohort (SHIP-2/Trend-0), with balancing weights

[‡]EQ-5D index value: fractional response model with average marginal effects

[§]EQ-5D subdomain impairments: Poisson regression

[‖]VanderWeele & Ding, Ann Int Med 2017
